# Exploring nursing home resident and their care partner priorities for care using the Action-Project Method

**DOI:** 10.1186/s12877-023-03863-9

**Published:** 2023-03-08

**Authors:** Andrea Gruneir, Matthias Hoben, Adam Easterbrook, Charlotte Jensen, Monica Buencamino, Jaclyn Tompalski, Stephanie A. Chamberlain, Sadaf Ekhlas, Gillian Bever, Ruth Murphy, Carole A. Estabrooks, Janice Keefe, Sheila Marshall

**Affiliations:** 1 Department of Family Medicine, Faculty of Medicine and Dentistry, College of Health Sciences, University of Alberta, 6-10 University Terrace, Edmonton, AB T6G 2T4 Canada; 2 Faculty of Nursing, College of Health Sciences, University of Alberta, 116 St. and 85 Ave, Edmonton, AB T6G 2R3 Canada; 3 Centre for Health Evaluation and Outcome Sciences, St. Paul’s Hospital, 588-1081 Burrard St., Vancouver, BC V6Z IY6 Canada; 4 Department of Sociology and Anthropology, Carleton University, B750 Loeb Building, 1125 Colonel By Dr, Ottawa, ON K1S 5B6 Canada; 5 Cumming School of Medicine, University of Calgary, 2500 University Dr NW, Calgary, AB T2N 1N4 Canada; 6 Department of Occupational Science and Occupational Therapy, University of British Columbia, T325-2211, Wesbrook Mall, Vancouver, BC V6T 2B5 Canada; 7 Translating Research in Elder Care (TREC) Research Program, Faculty of Nursing, College of Health Sciences, University of Alberta, 116 St. and 85 Ave, Edmonton, AB T6G 2R3 Canada; 8 Department of Family Studies and Gerontology and Nova Scotia Centre On Aging, Mount Saint Vincent University, 166 Bedford Highway, Halifax, NS B3M 2J6 Canada; 9 School of Social Work, University of British Columbia, Jack Bell Building, 2080 West Mall, Vancouver, BC V6T 1Z2 Canada

**Keywords:** Quality measurement, Care partner, Resident experience, Care relationships

## Abstract

**Background:**

Nursing home (NH) residents’ experiences are embedded within their relationships to others. Our objectives were to describe how residents and care partners (family or staff members) jointly construct, discuss, and act on care priorities.

**Methods:**

We used Action-Project Method, a qualitative method focused on action within social context. We recruited 15 residents and 12 care partners (5 family and 7 staff members) from 3 urban NHs in Alberta, Canada. Residents and care partners participated in a video-recorded conversation about their experiences in the NH, then individually reviewed the video-recording to add context to the conversation. Following transcription, preliminary narrative construction, and participant feedback, the research team conducted in-depth analysis to identify participant actions, goals, and projects, including those jointly shared by dyad members.

**Results:**

All participants’ intentions could be broadly described as “making time in the NH as good as possible” and projects were grouped into five categories: resident identity, relationships (both presence and absence), advocacy, positivity, and respectful care. Participants often raised issues of short-staffing as a significant barrier to respectful care. Care partners, especially staff, used positivity to redirect residents from difficult topics. Joint projects could be identified in some, but not all, cases.

**Conclusions:**

We found that maintaining a sense of identity, fostering relationships, and receiving respectful care were important to residents but that short-staffing created barriers. Methods to capture these aspects of the resident experience are needed but should not be influenced by care partners’ tendency towards positivity in resident interactions.

## Introduction

The quality of nursing home (NH) care has been of concern for decades. In response, there have been a series of attempts to define and monitor quality along with changes in oversight. In a comprehensive review of quality assessment in the U.S., Castle and Ferguson (2010) describe the evolution from a focus on care structures to care processes and ultimately clinical outcomes [1], consistent with Donabedian’s framework [2]. These changes in the focus of quality measurement were facilitated by the implementation of the Minimum Data Set, a comprehensive clinical assessment [3, 4]. While such measures have led to improvements on specific processes and outcomes [5–10], they have been criticized for being overly medical [11, 12].

Further, the rise of resident-directed care philosophies has not seen a commensurate rise is quality assessment derived from the resident experience [1]. This is despite the number of studies that have interviewed residents about their experiences and perceptions of quality [13, 14]. In a review of 24 such studies, Sion et al., (2020) found that residents experienced quality based on the physical and care environment, recognition of their individuality, and opportunities for social engagement [15]. In a meta-synthesis of studies of residents’ experiences in NHs, Vaismoradi and colleagues identified similar themes and drew particular attention to the difficulty of creating a “home-like” environment within the rigid institutional structure of NHs [16]. What these and others have shown is that residents often identify the non-technical aspects of care as central to their experience [17–19].

At the same time, relationship-centred care has begun to be recognized as the foundation for quality NH care. Relationships are embedded as the core of emerging quality frameworks such as the INDEXQUAL [20]. The INDEXQUAL framework conceptualizes quality from the resident’s perspective but with an explicit acknowledgement that that perspective is based on a co-created experience between the resident and care provider(s). The care provider role is not limited to NH staff and includes family members who often provide critical support for residents, including facility selection, direct care, and communication with staff [21–25].

The purpose of this study was to understand how NH residents and those involved in their regular care view and act on residents’ care priorities. We drew on the Contextual Action Theory and the Action-Project Method to develop and address our specific research questions: 1) How do NH residents and their care partners jointly construct, discuss, and act on care priorities within the setting? And 2) Can these joint actions be identified and described as joint projects? We chose this approach since it centres relationships and explores the co-actions of participants in creating the quality experience.

### Theoretical framework: Contextual Action Theory

Originally developed in counselling psychology, Contextual Action Theory (CAT) addresses behaviour (actions) within the social context and considers how these actions are both goal-oriented and intentional, even if not rational. CAT focuses on what people are doing to achieve their goals. Actions are considered from different perspectives: manifest behaviors, internal processes (cognitions and affect), and social meanings or goals [26]. Different levels of actions, specifically verbal and nonverbal behaviors, are linked to define the functional steps taken to reach a goal. CAT also considers systems of actions:

- short-term momentary actions,
- middle-term projects where linked actions are constructed around a common goal, and
- longer-term careers, represented by multiple projects throughout people’s lives [26, 27], where career refers to a longer-term process and not occupation [28].

CAT provides a useful approach for understanding how individuals act in relation to others and within their cultural context. In this study, CAT offers a framework for understanding the joint construction of and intentions for NH care.

## Methods

The Action-Project Method (A-PM) is a qualitative approach based on CAT [29]. The A-PM consists of an iterative process of data collection and reflection on participants’ actions and the meanings linked to those actions (see Table 1) [27, 30]. To our knowledge, this is the first use of the A-PM in NHs.

**Table 1:** Action-Project Method data collection and analysis process

Step	Who	Purpose
Data Collection		
Dyad Conversation	Nursing home resident and chosen care partner^a^	To discuss experiences within the nursing home and priorities for care
Video-Recall	Individual participant with research team member	To provide meaning and context to the video-recorded dyad conversation
Narrative Construction	Research team	To provide participants with a summary of the identified goals and actions
Narrative Review	Individual participant with research team member	To provide additional meaning, if any, to identified goals and actions; to provide participants with the opportunity to correct and/or provide approval to each the single and joint narratives
Data Analysis		
Within Case Analysis	Pair of team members (per dyad)	To create a case summary describing dyad’s intentional framework, goals, and projects (individual and joint)
•Code actions		
•Identify functional steps		
•Cross-check with narrative review		
Cross Case Analysis	Full team	To identify concordant and discordant findings, including common projects
•Compare cases by dyad composition		
•Compare cases by random assignment		

^a^Family member or staff

### Recruitment and participants

We used convenience sampling to recruit 17 residents from three large not-for-profit NHs in an urban area in Alberta, Canada. We contracted a licensed practical nurse (LPN) already employed in each NH to assist with recruitment. The LPN approached eligible residents and obtained consent for contact, if the resident was interested. Eligibility criteria were age 60 or older and ability to carry out a 10-min conversation in English. The LPN asked each resident to identify one person, either a family or staff member, with whom they would like to participate. Participants provided informed consent prior to the start of data collection. All data collection took place within the resident’s NH, mid-day or early afternoon Monday to Friday between January and May 2019. Three residents preferred to be interviewed on their own (two identified this at recruitment, one at the time of data collection). Of the remaining 14 residents, seven identified a family member (two adult children, five spouses) and seven identified a staff member (one paid companion, one recreation therapist, and five care aides). One resident died before data collection was completed and another was excluded because of audio-recording failure. The final sample included 15 residents, three participated alone, five with a family member, and seven with a staff member (Table 2).

**Table 2:** Participant information

Case Number	Resident Information	Care Partner Information	Length of Interview Minutes:Seconds	Additional Information
001	Female	Family Member (Adult Child)	13:16	
002	Female	None	39:15	
003	Male	Family Member (Spouse)	N/A	Excluded due to recording failure
004	Male	Family Member (Spouse)	6:31	
005	Female	None	21:50	Chose to participate alone at time of interview
006	Female	Staff Member (Care Aide)	8:37	
007	Male	None	28:43	
008	Female	Family Member (Spouse)	9:03	
009	Female	Family Member (Adult Child)	6:21	
010	Female	Staff Member (Paid Companion)	5:39	
011	Female	Staff Member (Recreation Therapist)	8:42	
012	Female	Staff Member (Care Aide)	5:49	
013	Male	Staff Member (Care Aide)	6:06	
014	Female	Staff Member (Care Aide)	5:06	
015	Female	Staff Member (Care Aide)	5:28	
016	Male	Family Member (Spouse)	6:29	

Does not include participant who died during data collection cycle

### Study team

This study was part of a larger mixed-methods project on the final year of life in NHs [31]. The research team included members with varied expertise of each the substantive and methodological areas (see Table 3 for details). The senior author, who has extensive expertise with the A-PM, oversaw all data collection and analysis and provided A-PM training to the team members. To prepare for data analysis, the team coded the same case and resolved discrepancies through discussion. Nearly all members of the research team had direct experience with NHs either through work, volunteer, training, or personal circumstances.

**Table 3:** Study team members and relevant expertise

Study Team Members	Expertise/Training
Team Leads (2)	•Nursing (PhD-trained)
	•Epidemiology and Health Services
Postdoctoral Fellow (1)	•Gerontology
Research Associates (2)	•Action-Project Method
	•Qualitative Data Analysis
Research Assistants (4)	•Sociology
	•Nursing
	•Public Health
	•Social Work
Senior Researchers (3)	•Action-Project Method and Family Studies
	•Nursing Home Care
	•Caregiving (Professional and Family/Friend)
Person with Lived Experience	•Family Caregiver

### Data collection

Data collection and analysis were iterative, and all participants were provided an opportunity to comment on initial results from their own data. The first step in data collection was the dyad conversation. Members of the data collection team explained to the participant dyad (resident and chosen care partner) that they were interested in hearing them discuss their experiences in the NH and ideals for care. Dyads were told to discuss any topic(s) that they thought they were important to their experience but were not prompted to discuss any particular aspects. Once the dyad conversation began, the research team members left the room but audio- and video-recorded the conversation; in this step, participant actions were recorded for observation. The research team members re-joined the dyad after approximately 5–10 min, stopped the recording, and then met separately with each dyad member for the second step in data collection, known as video-recall. In the video-recall, each participant watched their own dyad conversation with one research team member. The research team member would stop the recording at approximately one-minute intervals and ask the participant to elaborate on and/or provide further context to the conversation during that interval. Participants were asked what they were thinking and feeling during the dyad conversation as a means to capture their internal processes. The video-recall was also recorded. All recordings were professionally transcribed.

The research team then conducted a preliminary analysis to construct three narratives: one per dyad member and a joint narrative. The research team identified each participants’ goals and complementary behaviours; they also identified joint projects, a series of behaviours or actions intended to lead to a shared goal between dyad members. Within six weeks of the original meeting, the research team members presented each dyad member with their own narrative and the joint narrative to provide correction or confirmation. Each dyad member participated in the narrative review separately and this was again recorded. Once participants gave final approval of the narrative(s), data collection was considered complete. Typically, this process would be repeated over a longer time period [32, 33]; however, we chose a single cycle because of the particulars of the participants and the setting.

For the three participants without a care partner, we used a more typical unstructured interview process. In this case, the research team members, led by one member, would ask the participant about the experience of living in the NH and ideals for care. These interviews had a conversational style such that the participant had flexibility to guide the discussion and was not pressed to answer questions. These interviews were also recorded but there was no video-recall since the research team member was able to ask for elaboration and/or meaning as the interview progressed. Individual participant interviews were typically longer than dyad conversations at approximately 20–40 min. Individual interviews were followed-up with a narrative review.

### Data analysis

Following the narrative reviews, we conducted in-depth analysis (see Table 1), beginning with within case analysis (single dyad). First, for each dyad, a pair of research team members would independently identify an overall intentional framework, the broad goal emerging from the interaction, and code all relevant transcripts. Transcripts were time-coded to line up with the intervals in the video-recall sessions. Each sentence (or statement, depending on the participant’s speaking style) was coded using a standard coding list, as is typical for research using the A-PM, [34] developed previously by the senior author and modified for this study. Codes reflected the type of (verbalized) action (for example, question or agreement) and/or expressions of emotion (for example, expresses joy or expresses frustration). In this method, the action (not the topic) is the unit of analysis and the codes are used to capture the actions and their construction in participants’ language. For each coded minute of the conversation, the team would review the video-recall transcripts for additional information on intentions or meaning. Next, the research team members identified functional steps (steps taken towards goals) based on the codes, and then linked the functional steps to goals. This was done for each member of the dyad and for the dyad itself (resulting in individual and joint functional steps and goals). The functional steps and goals were repeatedly checked against the intentional framework, which was refined throughout coding. Next, each team member completed a case summary that included a description of the dyad’s context, interactional pattern, intentional framework, goals, and projects with attention to joint vs. independent goals and projects. The case summaries were cross-checked against the narratives and narrative reviews for consistency. Once completed, the assigned team members would review and discuss discrepancies. For individual interviews, a similar process was used but with goals inferred from the interview itself rather than using the video-recall. In the individual case summary, the interactional pattern described the participant’s interactions with the interviewer and only individual intentional framework and projects were included. We identified projects in the context of the conversation with the researcher. The full research team would discuss and reflect on each case [32, 33].

Once within case analysis was complete, we conducted cross case analysis, where cases were compared against one-another to identify both concordant and discordant findings, including common projects. We conducted two rounds of cross-case analysis. In the first, cases were grouped by dyad composition (family member, staff member, alone) so that we could see how cases compared against those with similar composition; in the second round, cases were randomly assigned so that we could identify differences and similarities across cases regardless of their composition. One team member documented all discussions.

### Rigor and trustworthiness

The rigor of the method is reflected in the use of multiple sources of information to access differing perspectives on the same action. We audio and videorecorded interactions between participants to capture their behaviours and then used the video-recall, which was also recorded, to access internal processes. All recordings were transcribed and checked for accuracy. We kept an audit trail of each dyad’s case and notes of all team meetings. For trustworthiness of interpretations, participants had the opportunity to make changes to the narrative summaries during the second meeting. Each case was analysed by two team members and then presented to the full team to prevent any one researcher from overly influencing the interpretations.

### Ethics

All methods were performed under relevant guidelines and regulations or under declaration of Helsinki.

## Results

We found that all participants were in some way working toward “making time in the NH as good as possible” but that the projects and specific functional steps varied. We identified five groups of projects that could be defined through actions related to: 1) defining the resident’s identity; 2) maintaining or building relationships; 3) advocating for self and others; 4) emphasizing positive feelings and expressions; and 5) centring respectful care. Although distinct, there was some overlap across groupings.

### Project group 1: defining identity

Both joint and individual projects frequently related to the resident’s identity and, in particular, (1) actions to maintain the residents’ pre-admission identity and (2) actions to promote an individualized identity. This was largely discussed in three ways. The first related to the difficulty of transitioning to the NH. Several residents, especially those with longer stays, reflected on their early time as being emotionally challenging with one resident stating:

“Getting used to that was really hard for me. I almost felt trapped.”(resident, 015).

Residents with long-stays, typically 2 or more years, also described moving on from that early state to one in which they had both greater acceptance of being in the NH and in finding ways to continue past interests. One long-stay resident described how she used her long-standing crafting hobbies to reach out to the new residents, with whom she empathized:

“I – I don’t know if I told you, I’m the Welcome Wagon…I try to go as soon as I can, because I know how I felt when I first came here.”(resident, 005).

The second way we identified such projects was in the use of recreational activities. This emerged as a link to pre-admission identities:

“…She’s a major gardener…They have a greenhouse out back, and the plants and planters and stuff. So she’s all happy with that.”(son, 009).

As well as a strategy for creating a niche among residents:

“I do a lot of work for the facility. Because I’m well enough.” (resident, 005).

Finally, residents and some family members spoke about the need to be seen as different or distinct from other residents. In some cases, this was described as purposely avoiding engaging with other residents:

“They are boring for me sometimes because the people who are playing horseshoes and Snakes and Ladders and stuff like that, their mental capacity isn’t that great.”(resident, 006).

In other cases, residents and family members put a strong emphasis on being capable to perform self-care and used this both as a way to distinguish themselves from other residents, who were seen as “needy” and as a lens through which they could be identified as helpful to staff:

“And I’ll get my – everything ready for them, and I’ll get washed. And so they don’t have to do it. I said ‘I’m ready.’ And she’s happy – and she puts me to bed.” (resident, 014).

### Project group 2: maintaining or building relationships

A number of projects centred on the relationship between the resident and others. This was identified in three ways, with two related to the presence of existing relationships and the third related to the absence of relationships. A consistent project among the dyads that included a family member was the maintenance of those familial relationships. This was characterized by regular visits, activities like having coffee together, and making small outings. This was exemplified in one case, where an adult son described the many ways in which he continued to connect with his mother through their shared interests:

“She’s a sports lover…I know she’s gonna be watching the hockey playoffs. So I just come and sit and watch – watch with her.” (son, 009).

Relationship projects with staff looked very different from those with family members. These more frequently could be seen as asymmetrical in that the resident would describe the relationship as close or that they preferred relationships with staff to relationships with other residents:

“I guess I have more friends among the staff than I do among the residents because you just have more in common with them …” (resident, 005).

However, during the video-recall or narrative review, staff members minimized their role in the resident’s life or described treating all residents the same.

“Hmmm…just a casual – it’s also the same with the other residents that we are looking after.” (staff member, 015).

Finally, although loneliness and social isolation were raised less often, in one individual interview, the resident spoke at length about these issues and the impact on his well-being and ability to cope with physical symptoms:

“…you resign yourself to the fact you got the pain and nothing you can do about it. But it’s like…I hate being depressed. I hate feeling lonely. I hate feeling scared. Feeling alone – not just lonely, but alone.” (resident, 007).

### Project group 3: advocating for self and others

Nearly all participants engaged in some form of advocacy, although this varied from individual incidents to efforts for system-level change. In several instances, the advocacy projects were described as a family or staff member calling attention to specific care needs, such as continence: “Well, I’ll talk to them again. I did before, because a couple of times – you said they didn’t do it.” (son,009) or skin care: “Yeah, they did not look after you, right? So…that’s why I keep saying that– please apply the cream. And make sure everything is clear, right?” (staff member, 011), or respecting resident privacy: “And we’ve had a few instances with care personnel who assist the nurses, who have come in and …. Basically not observed the motto that the room is the resident’s room. And it’s their home” (daughter, 001).

In a few cases, residents described advocating on their own behalf in terms of learning how to have their requests met: “I didn’t know how much it bothered me, the privacy thing, until I started getting upset here about it. So…I’m gonna talk to the floor manager about it.” (resident, 002).

For two of the dyads, advocacy was directed to system change. In one case, the resident often used “we” to express a collective experience while the dyad partner (paid companion) based comments on observations of resident treatment and staffing issues; both dyad members spoke at length on the need for greater mental health care and attention to relational care:

“And it’s sad for you to have to see someone suffer. And it’s also sad that you know, that there’s no help for that person.” (staff member/paid companion, 010).

In another case, a resident and spouse raised issues of safety and well-being that they saw as the direct result of management and staffing problems:

“Yeah. So, they’re not understanding you. And you’re not understanding them. And so, that’s a – a grave concern for health and safety.” (spouse, 016).

This dyad took their advocacy beyond the individual facility:

“And, you know, like I—I wrote to the MLA and I said ‘If you can imagine a foster parent putting a baby on a bed and you know the baby fell off the bed. Well – and gets hurt. Well, the first time you might get away. But the second time, you’re up for child abuse. This is elder abuse.’ And I had to fight.” (spouse, 016).

### Project group 4: emphasizing positivity

In nearly all cases, projects could be characterized through positivity, specifically as attempts to focus on or reframe the conversation to positive aspects of life in the NH. In one individual interview, the resident deflected questions about health or care issues and focused on how she kept busy and helping others:

“It’s better if you can ignore the bad things…” (resident, 005).

In other cases, positivity was used to redirect residents from more difficult topics to things that they enjoyed. In dyads with staff members, they often took on an interviewer role and attempted to guide conversation to light topics and interrupted or glossed over more serious concerns raised by the resident. For example, one resident was trying to discuss his electric wheelchair being broken but the staff member repeatedly re-directed the conversation:

Resident: “And…it was a lot easier when my chair was ah…”.

Staff member: “So you have one daughter only, right? Did she visit you… regularly..or…?”.

Resident: “No, not – not enough.”

Staff member: “Oh, anyway…you have family here in extended care, right? And then…so how about the care here?”(011).

In one case, it was not until the resident repeatedly raised concerns about treatment by certain staff that the participating staff member acknowledged her concerns: “Our job here as a nursing aide, we just want – the resident will be happy, and respect, and then, ask whatever they want.” (staff member, 015). During the video-recall portion, one staff member directly acknowledged diverting the resident from difficult topics, including his wife’s death:

“Every time we talk about the family, about his wife, he cries. Yeah, because he said ‘I love so much my wife’ yeah. That’s why sometimes I don’t talk about it. Yeah, and the we try to…divert.” (staff member, 011).

This finding was not restricted to staff members. In one spousal dyad, each member tried to protect the other by focusing on positive topics. In another spousal dyad, the spouse minimized the resident’s feelings about living in the NH:

“But … that’s normal, because I think we – most people would prefer to be at home, but they can’t, right? So, that’s why they’re here. And unfortunately, that’s… that’s the way life is.” (spouse, 008).

### Project group 5: centring respectful care

Several participants discussed aspects of interpersonal treatment and care, and while they differed in specifics, they shared a common project around the promotion of respectful care. These projects often touched on resident autonomy, privacy, and individualized care needs. One resident exclaimed “treat me with some decency” (resident, 015) when describing her interactions with staff. Another resident described instances of staff entering her room and asking her to guess who it was despite their knowledge of her vision limitations.

“Um, so when I asked who it was, she said, “Guess.” And that irritates me….Because – especially since she knows I can’t see.” (resident, 002).

In a number of cases, time restrictions due to short-staffing were raised as a specific barrier to respectful care. Several of the residents and family members spoke about this directly:

“And the other…the other part is, there’s many, many good staff here. But they are way too overworked.” (spouse, 016).

As well as the implications for residents, including their need to accommodate staff routines (“I’ve had to make an effort to fit into their schedule” (resident, 002)). Time restrictions were so widely known, one dyad discussed the issue as something that becomes a resident worry:

Resident: “We’re always short staff.”

Companion: “And you have to hear about it. So you’re here and the – the nurses can’t have their problem and keep it separate.”(010).

Participants also spoke about the reluctance of staff to take on certain tasks, especially in circumstances of being short staffed (“Cause that’s another burden on your social – like… if it’s your day to go out with me and someone come in, ‘Well, you’re too heavy, I don’t want to give you a shower today.’ How is that supposed to reflect on your mental health?” (companion, 010)) and the eventual effect on resident comfort in asking for assistance (“That can build up, too… where the resident stops asking for things. Because he or she is afraid to offend the person or – or inconvenience a person.” (resident, 002)).

### Joint projects

While we were able to able to identify common themes across the projects, we found identifying joint projects was much more difficult because in several cases, participants’ actions were too disparate. Joint projects were more easily identified in dyads that included family members. This was particularly salient in one resident-adult child dyad where both members talked about spending time together and engaging in their long-standing interests, as well as, in a spousal dyad, where both members spoke about their advocacy for change. It was more difficult to identify joint projects in dyads with staff members, who often kept the focus on the resident, even during the video-recall. Further, despite near daily interactions, sometimes over long periods of time, residents and staff members reported having little time to engage beyond completing care tasks. In other cases, joint projects were difficult to identify because residents often described relationships with staff as central to their experience whereas staff discussed their efforts to support all residents without favouritism. In other cases, regardless of the dyad partner, the use of positivity to steer or frame conversations forced the resident to engage in specific ways. This was evident even in dyads where both participants shared a clear goal but approached it with different strategies. In one example, a spousal dyad shared the goal of coming to terms with the resident’s life in the NH but where the resident sought understanding, the spouse focused on the benefits of the NH.

## Discussion

In this study, we used CAT to understand how NH residents and their care partners acted on their care priorities and whether those actions could be viewed as joint projects. We found that all participants – both residents and care partners – were working towards making the resident’s time in the NH as enjoyable as possible but this was acted upon in different ways. Among our participants, projects could be organized into five groups: resident identity, relationships, advocacy, positivity, and respectful care. While some dyads, exhibited clear joint projects, these were not consistently observed, especially among those dyads that included staff members, who maintained their professional role even within the dyad conversation.

Not surprisingly, the project groupings that we identified are consistent with prior research on the resident experience and consistent with the Senses Framework [35], which describes the core necessities for good care. Adjusting to life in the NH and the accompanying changing roles and challenges to personal identity, maintaining long-term relationships and establishing new ones, and staying meaningfully engaged have all been described by others as critical to creating a quality NH experience [15, 16, 36]. Also consistent were the advocacy projects, which consistently reflected issues with short-staffing and the effects on resident care and the ability to develop meaningful relationships [37].

By using the A-PM, a method explicitly focused on interactions between close individuals, we achieved two items that could not be using traditional interview-based methods. First, by recruiting residents and care partners, the A-PM creates space for both actors to demonstrate their goals for quality care and how those are enacted. This is reflective of a relationship-centred care philosophy, which considers quality a shared experience by residents, staff, and families and friends [20, 38]. Our findings also reinforce the differing roles that family and staff play in constructing the care experience with residents, even as they work toward common goals. Of those residents who chose to participate alone, their rationale for doing so and their individual projects often reflected how relationships factored into their care. Second, the use of positivity by care partners to steer interactions with residents and reframe conversations could not have been identified using narrative interviews alone. Although it was clear that care partners meant well, their excessive positivity meant that residents had little opportunity to freely express themselves, even when asked to do so. While such paternalistic behaviours have been described before, [39] our findings demonstrate the impact that incessantly positive interactions can have on residents’ ability to fully express themselves and have their needs met, and that positivity seemed to diminish resident engagement in joint projects. As this is the first study to use A-PM in NHs, future research should focus on better refining the methods [40] for this setting and more deeply exploring actions related to specific aspects of care, both of which would further clarify where this method offers value beyond narrative interviews.

While participants did discuss physical symptoms and disability, these were often described within the context of how residents were treated during the process of care. Issues like timeliness of staff response, staff attitude, and maintenance of privacy (all aspects of respectful care) were more frequently described as part of the resident experience than any physical concern. Even discussions of mental health focused on the support aspects of care rather than on medical management. Further, there was a strong emphasis on relational care and the need for staff to have time to make residents feel worthy of care, and not just a series of tasks. Quality, as determined by our usual structure-process-outcome measures, has not been found to be correlated with experience measures [17] nor does it reflect culture change [41]. How to measure and report on the resident experience, in particular the relational aspects, remains a challenge. Emerging strategies to assess resident experienced care, such as the “Connecting Conversations” narrative method, [42, 43] show promise but require additional study for successful implementation given the reality of the NH environment. At the same time, key structure-process-outcomes measures must continue to be collected to ensure technical care quality [6], which cannot be overlooked, but should not detract from a relation-centred care experience of residents, families, and staff. More importantly, though, any such measures need to be actionable – that is reporting on poor relational care or staff time pressures would need to be met with the same attention as clinical outcomes, such as antipsychotic use without indication, and would require a fundamental shift in our approaches to quality improvement and practice. Such considerations are important if NHs are to fulfill their potential to meet this vulnerable population’s clinical, social, and emotional needs.

### Limitations

There are limitations to consider. First, we did not include residents with advanced cognitive impairment, whose experience may differ in important ways. Second, participants largely agreed during their narrative reviews, making it difficult to know if our analyses fully captured participants’ goals and projects or if they felt the need to agree with us. Third, participants often had a hard time getting into the conversation and in many dyads, the care partner started by questioning the resident before a true conversation began. Consequently, in some cases, the research team may have ended the conversation too early. Fourth, staff participants were clearly under significant time pressures, which influenced how much time they could spend in data collection. Finally, we used only a single data collection cycle for reasons described earlier; however, this combined with limited longitudinal research on the resident experience means that we have little understanding of how resident and care partner goals, and their subsidiary actions, may change from admission to death. Future research on the effect of time on care quality and the methods to study this are needed.

## Conclusions

In this study, we sought to understand the resident experience within the context of their relationships with family and staff. Our findings reinforce the importance of maintaining a sense of identity, fostering relationships, and receiving respectful care in the resident experience. Limits on staff time – in particular as the result of short-staffing – were seen as a substantial barrier to both respectful and appropriate care, and underlay the discussions on advocacy at both the individual and system levels. Routine collection and reporting of quality measures need to be able to incorporate these elements to better capture the full resident experience; however, doing so, especially with methods that are not vulnerable to care partners’ tendencies for positivity in their interactions with residents, continues to be a challenge.

## Data Availability

The data collected and analyzed for this study are not publicly available because they include video and audio of participants and personal information (including first names). Data can be made available from the corresponding author upon reasonable request.

## Abbreviations

A-PM: Action Project Method
CAT: Contextual Action Theory
LPN: Licensed Practical Nurse
MLA: Member of Legislative Assembly
NH: Nursing Home
